# Supplementary figures and images for: Development and Biological Characterization of Cancer Biomimetic Membrane Nanovesicles for Enhancing Therapy Efficacy in Human Glioblastoma Cells
Source: Nanomaterials (Basel). 2024 Nov 5;14(22):1779. doi: 10.3390/nano14221779 (PMC11597144; doi:10.3390/nano14221779)

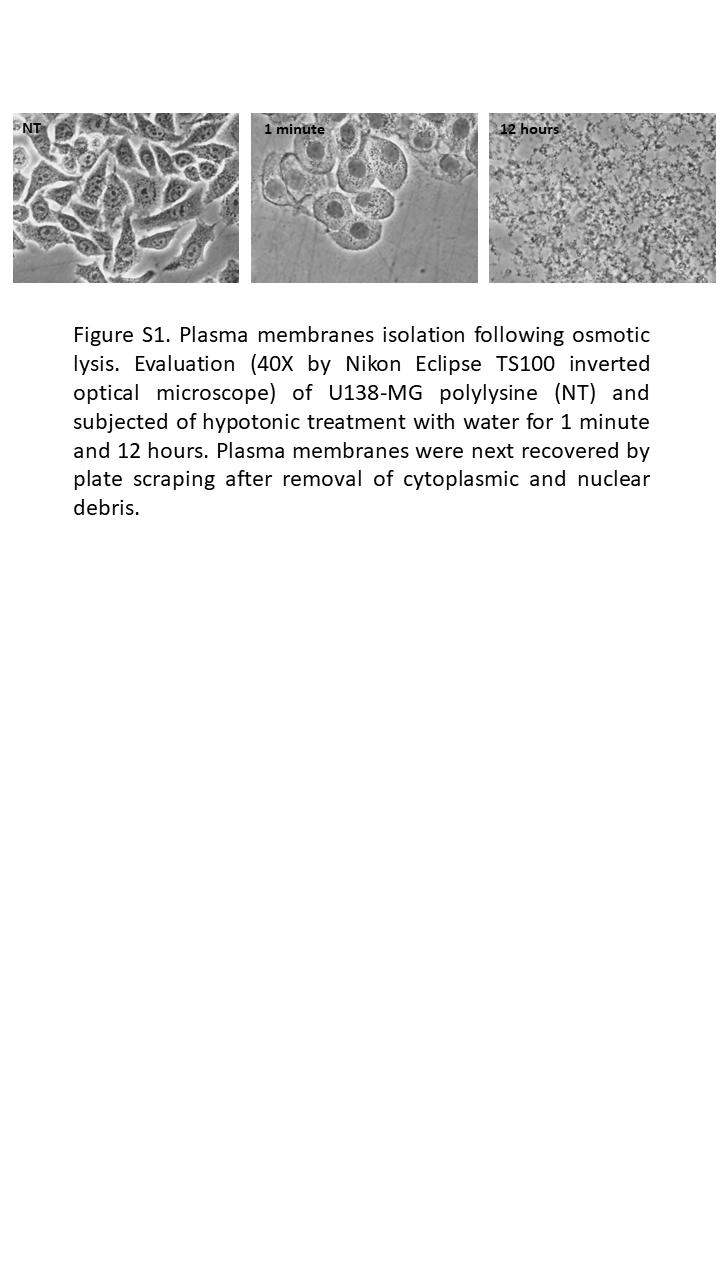

Supplement: Supplementary file 1 [file nanomaterials-14-01779-s001.zip › Figure S1.tif]

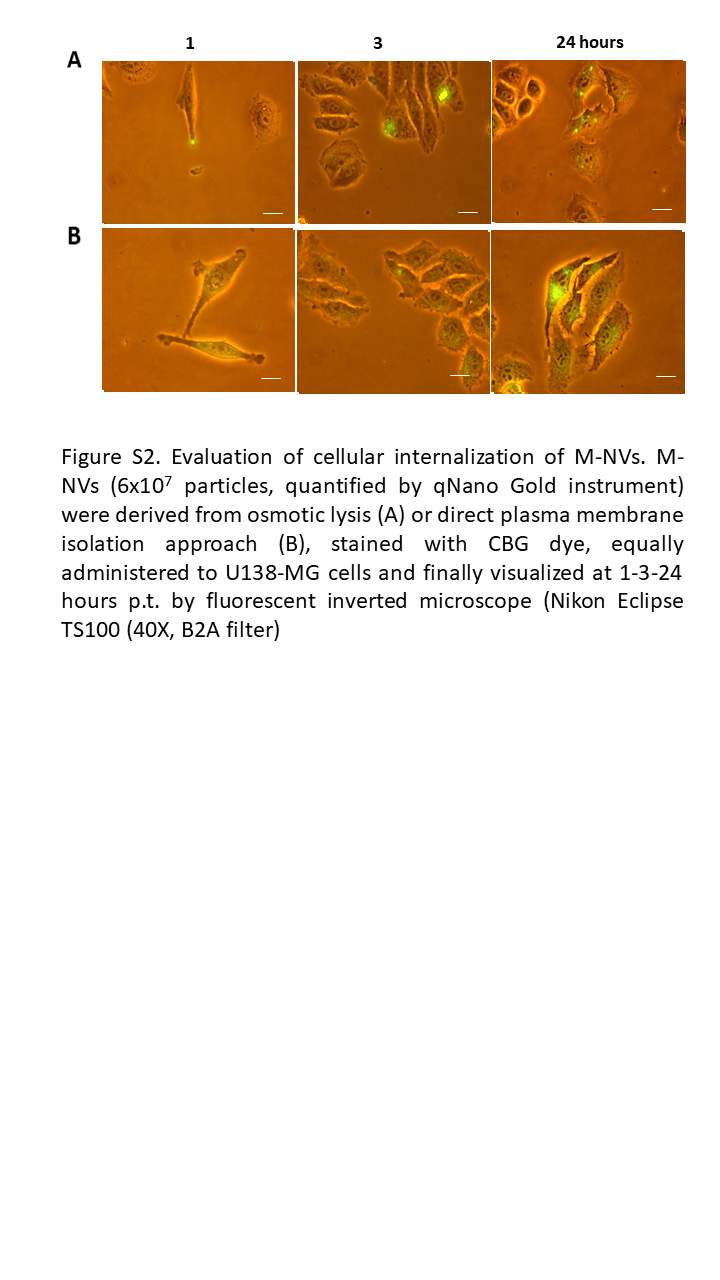

Supplement: Supplementary file 1 [file nanomaterials-14-01779-s001.zip › Figure S2.tif]

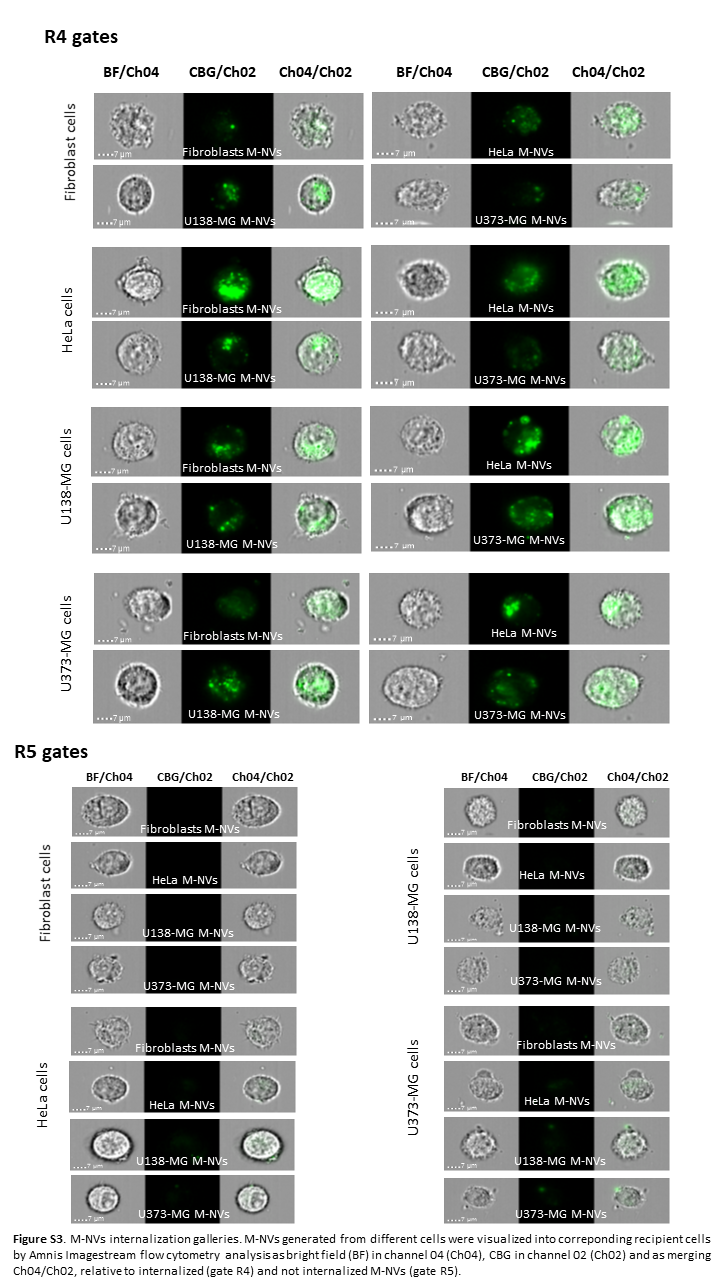

Supplement: Supplementary file 1 [file nanomaterials-14-01779-s001.zip › Figure S3.tif]

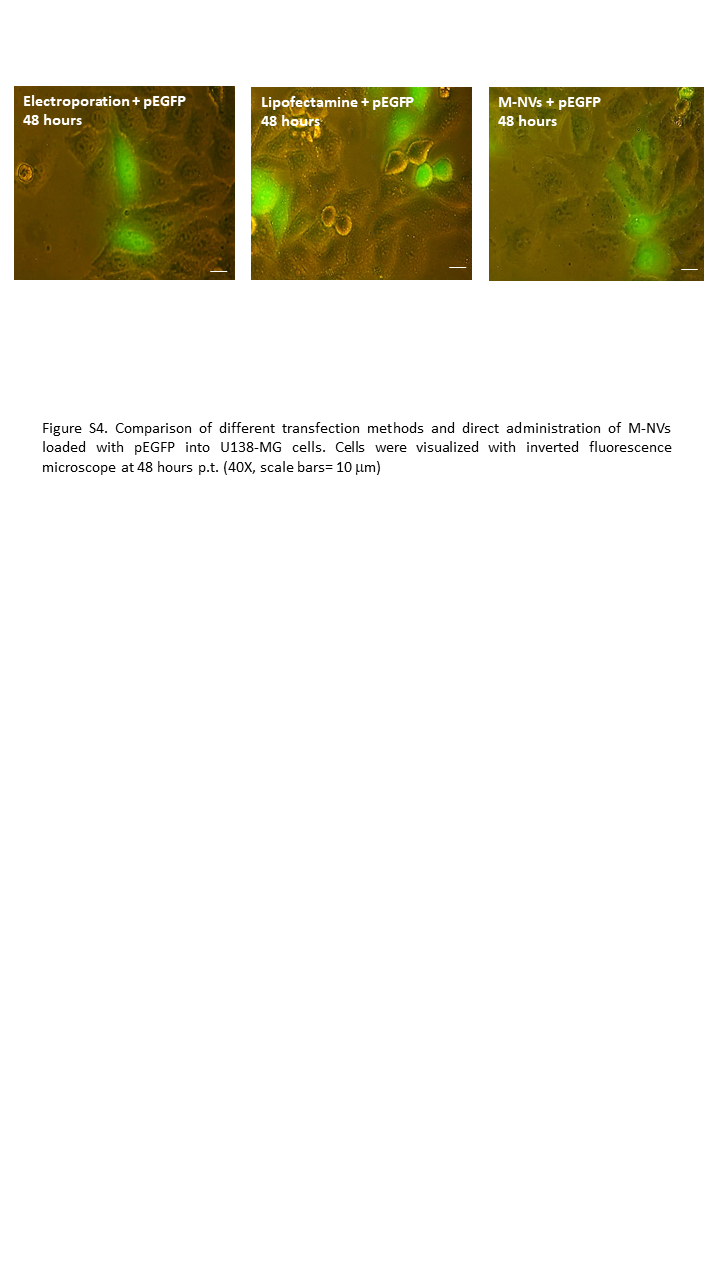

Supplement: Supplementary file 1 [file nanomaterials-14-01779-s001.zip › Figure S4.tif]

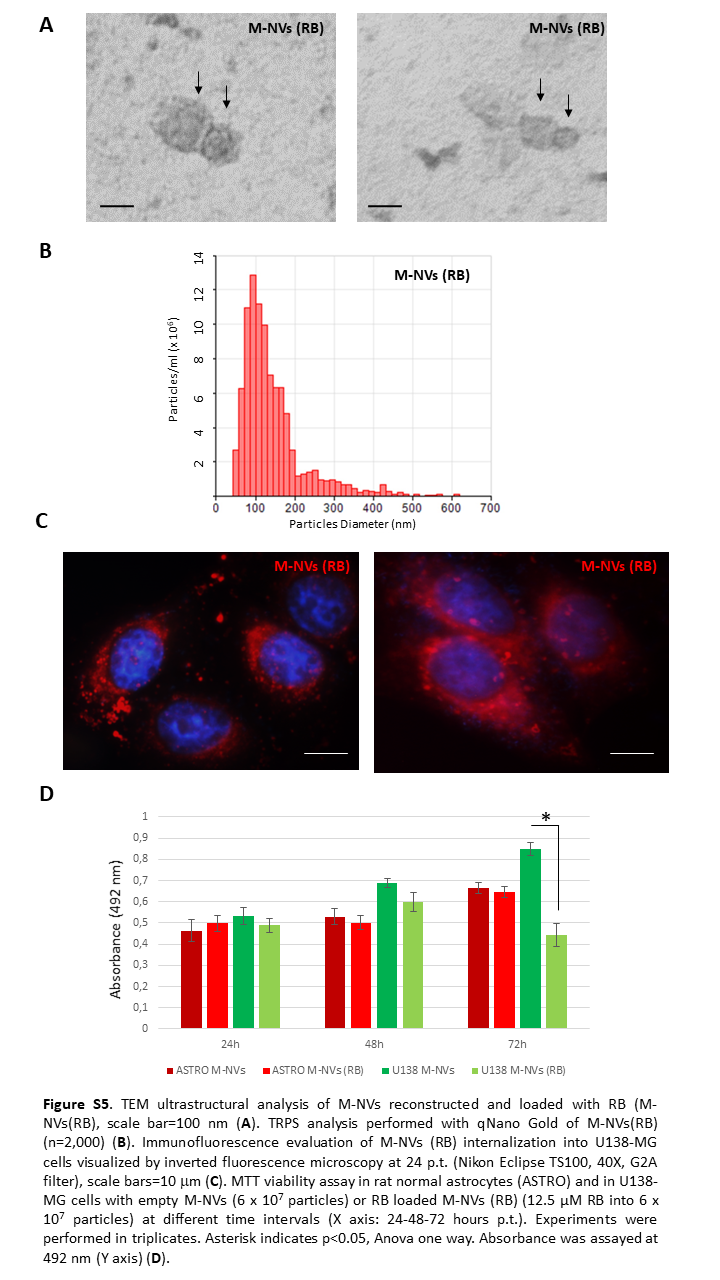

Supplement: Supplementary file 1 [file nanomaterials-14-01779-s001.zip › Figure S5.tif]
